# DRB1*03:01 Haplotypes: Differential Contribution to Multiple Sclerosis Risk and Specific Association with the Presence of Intrathecal IgM Bands

**DOI:** 10.1371/journal.pone.0031018

**Published:** 2012-02-17

**Authors:** Emilio G. de la Concha, María L. Cavanillas, M. Carmen Cénit, Elena Urcelay, Rafael Arroyo, Óscar Fernández, José C. Álvarez-Cermeño, Laura Leyva, Luisa M. Villar, Concepción Núñez

**Affiliations:** 1 Department of Clinical Immunology, Hospital Clínico San Carlos, Instituto de Investigación Sanitaria San Carlos (IdISSC), Madrid, Spain; 2 Department of Neurology, Hospital Clínico San Carlos, Instituto de Investigación Sanitaria San Carlos (IdISSC), Madrid, Spain; 3 Department of Neurology, Clinical Neurosciences Institute, Hospital Regional Universitario Carlos Haya, Málaga, Spain; 4 Department of Neurology, Hospital Ramón y Cajal, Madrid, Spain; 5 Research Laboratory. Clinical Neurosciences Institute, Hospital Regional Universitario Carlos Haya and Fundación IMABIS, Málaga, Spain; 6 Department of Immunology, Hospital Ramón y Cajal, Madrid, Spain; University Hospital La Paz, Spain

## Abstract

**Background:**

Multiple sclerosis (MS) is a multifactorial disease with a genetic basis. The strongest associations with the disease lie in the Human Leukocyte Antigen (HLA) region. However, except for the DRB1*15:01 allele, the main risk factor associated to MS so far, no consistent effect has been described for any other variant. One example is HLA-DRB1*03:01, with a heterogeneous effect across populations and studies. We postulate that those discrepancies could be due to differences in the diverse haplotypes bearing that allele. Thus, we aimed at studying the association of DRB1*03:01 with MS susceptibility considering this allele globally and stratified by haplotypes. We also evaluated the association with the presence of oligoclonal IgM bands against myelin lipids (OCMB) in cerebrospinal fluid.

**Methods:**

Genotyping of *HLA-B*, *-DRB1* and *-DQA1* was performed in 1068 MS patients and 624 ethnically matched healthy controls. One hundred and thirty-nine MS patients were classified according to the presence (M+, 58 patients)/absence (M−, 81 patients) of OCMB. Comparisons between groups (MS patients vs. controls and M+ vs. M−) were performed with the chi-square test or the Fisher exact test.

**Results:**

Association of DRB1*03:01 with MS susceptibility was observed but with different haplotypic contribution, being the ancestral haplotype (AH) 18.2 the one causing the highest risk. Comparisons between M+, M− and controls showed that the AH 18.2 was affecting only M+ individuals, conferring a risk similar to that caused by DRB1*15:01.

**Conclusions:**

The diverse DRB1*03:01-containing haplotypes contribute with different risk to MS susceptibility. The AH 18.2 causes the highest risk and affects only to individuals showing OCMB.

## Introduction

Multiple sclerosis (MS) is a chronic inflammatory disease which affects the central nervous system and it is characterized by myelin destruction, axonal degeneration and progressive neurological dysfunction. MS shows a complex etiology, which comprises the interplay of genetic and environmental factors. A clear and well established influence of the Human Leukocyte Antigen (HLA) allele DRB1*15:01 has been shown in almost all the populations studied, pointing to this allele as the main risk factor contributing to disease susceptibility. However, it is known that other HLA genetic variants influence disease development, but the additional genetic contribution of this region is far to be understood. Although the special characteristics of this region and the very probable existence of epistatic interactions between different HLA risk alleles [1], [2] hampers the study of this region, small steps can be taken trying to elucidate this problem. A relevant issue to deal with is the one concerning DRB1*03:01. This allele has been consistently associated with high MS risk in Sardinia [3], [4], but heterogeneous data exist regarding the effect of DRB1*03:01 in other populations [1], [3], [5], [6], [7], [8].

HLA peculiarities include the existence of specific allele combinations over large regions which have been conserved across generations and conform the so-called ancestral haplotypes (AH). The DRB1*03:01 allele is present in two AH: 18.2 and 8.1, i.e., it can be found in two different haplo-specific contexts. Besides, this allele can be found within non-specific allelic combinations, including partially-conserved 18.2 and 8.1 AH fragments, thereafter called non-conserved haplotypes. The frequency of those DRB1*03:01-containing haplotypes varies greatly depending on the geographical location. Thus, in populations of Northern Europe DRB1*03:01 appears preferentially in the AH 8.1, but it does in the AH 18.2 in Sardinia. According to this, differences in the MS risk conferred by those haplotypes could be underlying the heterogeneity observed in the previously published studies focused in the DRB1*03:01 allele.

In the Spanish population, our research group described an association between MS susceptibility and one polymorphism located in the promoter of the tumor necrosis factor-alpha (*TNF*) gene, rs1800750 (*TNF* -376) [9], [10]. Later, association with *TNF* rs1800750 was also found in a subgroup of Spanish MS patients characterized by the presence of oligoclonal IgM bands against myelin lipids (OCMB) restricted to cerebrospinal fluid (CSF) [11], which has been described as conditioning an aggressive MS course. In both cases, the susceptibility allele was rs1800750_A, which is almost exclusively present on the AH 18.2. Of note, in Spain, like in other populations of South Europe, the three subsets of DRB1*03:01-bearing haplotypes (AH 18.2, AH 8.1 and non-conserved ones) appear approximately at a similar frequency. This situation enables us to investigate whether those subsets contribute in a similar manner to MS susceptibility.

In the present work, we aimed at studying the role of DRB1*03:01 in relation to MS risk and production of oligoclonal IgM against myelin lipids, with special attention to the putatively different contribution of the different haplotypes bearing that allele.

## Methods

### Ethics Statement

This study was approved by the ethical committees of the participant hospitals (Comité Ético de Investigación Clínica (CEIC) del Hospital Clínico San Carlos, CEIC del Hospital Ramón y Cajal and CEIC del Hospital Universitario Carlos Haya). Samples were obtained after obtaining written informed consent.

### Subjects

We studied 929 MS patients diagnosed following the Poser criteria [12] and 624 ethnically matched individuals without autoimmune diseases as controls. All individuals were Spanish of Caucasian ancestry. Samples were consecutively recruited at both Hospital Clínico San Carlos (Madrid) and Hospital Universitario Carlos Haya (Málaga). Population stratification is not present in our samples, since reduced genomic control inflation factor was observed after genotyping more than 200,000 SNPs.

To study the influence of DRB1*03:01 on the presence of OCMB, we studied 139 independent MS patients recruited at the Hospital Ramón y Cajal (Madrid). All these patients showed a relapsing-remitting disease; a total of 58 patients showed OCMB in CSF (M+ group) and 81 lacked them (M− group).

### HLA Genotyping

DNA was extracted from fresh peripheral blood leukocytes by a “salting out” procedure. *HLA-B*, -*DRB1* and -*DQB1* were typed by polymerase chain reaction-sequence-specific oligonucleotide (PCR-SSO) following manufacturer recommendations (Tepnel Lifecodes Corp., Stamford, CT, USA). The presence of the DRB1*15:01 allele was also tested by TaqMan technology using the highly correlated SNP rs3135388 (Applied Biosystems Inc., Foster City, CA, USA).

AH 18.2 and 8.1 were assessed by genotyping of *HLA-B*, -*DRB1* and -*DQB1*. Additionally, the *TNF* singe nucleotide polymorphisms −308 (rs1800629) and −376 (rs1800750) and the microsatellites *TNFa* and *TNFb* were also ascertained. *TNF* polymorphisms were typed as previously described [9], [13].

### IgM detection

Detection of the lipid-specific oligoclonal IgM bands was performed by isoelectric focusing and immunoblotting as previously described [14].

### Statistical analysis

HLA haplotypes were estimated using the EM (Expectation-Maximization) algorithm implemented in the Arlequin software.

Comparisons between groups were performed with the chi-square test or the Fisher's exact test when proper (expected values below 5) using the statistical package EpiInfo v5.00 (CDC, Atlanta, USA). Heterogeneity between groups was evaluated with Review Manager (RevMan) 5.0 software (Copenhagen: The Nordic Cochrane Centre, The Cochrane Collaboration, 2008).

## Results

No heterogeneity was observed between samples collected in Madrid and Málaga, therefore, pooled data are presented.

The frequency of the different DRB1*03:01-containing haplotypes in MS patients and in controls is shown in Table 1. Patients were stratified according to the presence/absence of the DRB1*15:01 allele risk, but no differences were found between those two MS groups when DRB1*03:01 data were analysed; therefore, all MS patients were combined (column “total” of Table 1) for case-control comparisons.

**Table 1:** Frequency of the different DRB1*03:01-containing haplotypes in DRB1*15:01 positive (+) and negative (−) multiple sclerosis (MS) patients and in controls; and case-control study for the total MS patients.

	MS PATIENTS						CONTROLS		MS PATIENTS vs. CONTROLS	
	DRB1*15:01 (−)		DRB1*15:01 (+)		Total					
	n	%	n	%	n	%	n	%	p	OR (95% CI)
DRB1*03:01										
**All**	233	19.5	57	17.2	290	19.0	167	13.4	7.1*10^−5^	1.52 (1.23–1.88)
**AH 18.2**	88	7.4	24	7.2	112	7.3	49	3.9	1.3*10^−4^	1.94 (1.36–2.78)
**AH 8.1**	60	5.03	17	5.1	77	5.05	53	4.3	0.23	1.25 (0.86–1.81)
**Non-conserved**	85	7.1	16	4.8	101	6.6	65	5.2	0.072	1.34 (0.96–1.87)
**AH 8.1+Non-conserved**	145	12.1	33	9.9	178	11.7	118	9.5	0.027	1.32 (1.02–1.70)
**TOTAL NUMBER**	1194		332^1^		1526^1^		1248			

% are referred to the total number of haplotypes analysed in each group (last row).

AH 18.2 includes haplotypes carrying DRB1*03:01, DQB1*02:01, *TNF -376A*, *TNF a1b5* and B*18. AH 8.1 includes haplotypes carrying DRB1*03:01, DQB1*02:01, *TNF -308A*, *TNF a2b3* and B*8. Haplotypes with all the remaining allelic combinations in those loci or markers are included as “non-conserved” haplotypes.

1 Excluding the HLA-DRB1*15:01-containing haplotypes.

First, we investigated the possible role of DRB1*03:01 in MS susceptibility and we observed a highly significant effect: OR = 1.52, 95% CI 1.23–1.88, p = 7.1*10^−5^. Since AH 18.2 and AH 8.1 appear at a similar frequency in the Spanish controls (around 30% of all DRB1*03:01), we could evaluate the possible different contribution of those two haplotypes in disease risk. We started with AH 18.2 analysis, which showed a significant association and higher risk than when considering all DRB1*03:01-bearing haplotypes: OR = 1.94, 95% CI 1.36–2.78, p = 1.3*10^−4^ (Table 1). Due to its key role in MS risk, 18.2 haplotypes were removed from subsequent analysis in order to ascertain a putative effect of other AHs. We examined the influence of AH 8.1, but a significant result was not obtained. Similarly, no association was found when considering the non-conserved DRB1*03:01 haplotypes. However, since statistical power limitations could be affecting these results, we combined AH 8.1 and non-conserved DRB1*03:01 haplotypes data (given their lack of heterogeneity: p = 0.76; I^2^ = 0%) and then a significant result emerged (p = 0.027, OR = 1.32, 95% CI 1.02–1.70), pointing to a contribution of DRB1*03:01 carrying haplotypes others than AH 18.2 to MS risk, as seems to be deduced from the existing literature. To obtain a more accurate estimation of the specific effect caused by AH 18.2, we excluded AH 8.1 and non-conserved haplotypes from the initial analysis and we obtained an OR = 2.00, 95% CI 1.40–2.87 (p = 6.4*10^−5^). Of note, heterogeneity was detected between AH 18.2 and the remaining DRB1*03:01 haplotypes: p = 0.05, I^2^ = 74%, supporting the mentioned stronger effect on MS risk of this AH.

Next, we studied the involvement of DRB1*03:01 in the presence of OCMB in MS patients (Table 2). No differences were observed between patients stratified by the presence (M+ group)/absence (M− group) of OCMB when considering all DRB1*03:01 haplotypes. However, a different scenario emerged after individual analysis of the different DRB1*03:01 haplotypes. The previously observed effect for AH 18.2 was exclusively present when studying M+ patients (p = 0.0088 M+ vs. M−; p = 0.0055 M+ vs. controls). No differences seemed to exist between M+ and M− for DRB1*03:01 haplotypes others than the AH 18.2.

**Table 2:** Frequency of the different DRB1*03:01-containing haplotypes in multiple sclerosis (MS) patients stratified by the presence/abscence of oligoclonal IgM against myelin lipids (M+/M−) and in controls; and comparisons between groups.

	MS PATIENTS				CONTROLS		M+ vs. M−	M+ vs. Controls	M− vs. Controls
	M+		M−				p, OR (95%CI)	p, OR (95%CI)	p, OR (95%CI)
	n	%	n	%	n	%			
DRB1*03:01									
**All**	19	20.4	20	15.2	167	13.4	0.30, 1.44 (0.68–3.04)	0.058, 1.66 (0.94–2.90)	0.57, 1.16 (0.68–1.96)
**AH 18.2**	10	10.8	3	2.3	49	3.9	0.0088, 5.05 (1.23–29.24)	0.0055, 2.98 (1.36–6.39)	0.27, 0.59 (0.12–1.88)
**AH 8.1**	2	2.2	6	4.5	53	4.3	0.48, 0.50 (0.05–2.93)	0.55, 0.54 (0.06–2.17)	0.84, 1.09 (0.38–2.62)
**Non-conserved**	7	7.5	11	8.3	65	5.2	0.94, 0.96 (0.32–2.84)	0.19, 1.57 (0.59–3.60)	0.15, 1.63 (0.79–3.30)
**AH 8.1+ Non-conserved**	9	9.7	17	12.9	118	9.5	0.61, 0.80 (0.31–2.03)	0.77, 1.11 (0.51–2.37)	0.23, 1.39 (0.78–2.46)
**TOTAL NUMBER**	93		132		1248				

% are referred to the total number of haplotypes analysed in each group (last row).

AH 18.2 includes haplotypes carrying DRB1*03:01, DQB1*02:01, *TNF -376A*, *TNF a1b5* and B*18. AH 8.1 includes haplotypes carrying DRB1*03:01, DQB1*02:01, *TNF -308A*, *TNF a2b3* and B*8. Haplotypes with all the remaining allelic combinations in those loci or markers are included as “non-conserved” haplotypes.

HLA-DRB1*15:01-containing haplotypes in MS patients were removed from the analysis.

Since the two groups of MS patients studied (Table 1 and Table 2) constitute independent samples, we combined their data to increase statistical power and a significant association was observed when considering the DRB1*03:01 non-conserved haplotypes: p = 0.037, OR = 1.39, 95% CI 1.01–1.93.

We also evaluated the effect of the DRB1*15:01 allele in M+ and M− patients. A similar risk was observed in both groups of patients, also similar to the risk observed when considering the overall MS patients: OR = 2.56, 95% CI 1.99–3.29 (allelic risk). This effect is similar to the one caused by the AH 18.2 in M+ patients (heterogeneity: p = 0.69; I^2^ = 0%) and significantly higher to the risk conferred by the non-conserved DRB1*03:01 haplotypes (heterogeneity: p = 0.003; I^2^ = 89%).

## Discussion

Last years have witnessed an increasing number of genetic regions involved in MS susceptibility; however, the strongest association signals with disease risk remain in the long-known HLA region. Nevertheless, except for the well-established effect of HLA-DRB1*15:01, genetic influence at this region remains elusive.

We studied the risk conferred by the different DRB1*03:01-containing haplotypes (AH 18.2, AH 8.1 and non-conserved haplotypes) in relation to MS susceptibility and, specifically, to the presence of intrathecal IgM against myelin lipids restricted to CSF in MS patients. Our results indicate that patients bearing the AH 18.2 show an increased risk to produce intrathecal IgM (OR = 2.98 95% CI 1.36–6.39) and this effect seems to be independent of the presence of HLA-DRB1*15:01. The strong effect caused by this haplotype, together with the considerable percentage of M+ individuals among MS patients (around 40%), makes that a susceptibility signal emerges also when considering the overall MS patients independently of the presence/absence of OCMB, although, obviously, causing an apparently lower risk.

This finding could explain the heterogeneous results regarding HLA-DRB1*03 previously published. The percentage of AH 18.2 from the total HLA-DRB1*03 and the percentage of M+ individuals from the total MS patients will determine obtaining a significant result. Moreover, the situation is probably more complicated. Although statistical power limitations preclude a conclusive interpretation of data regarding DRB1*03 haplotypes others than 18.2, it seems that at least the non-conserved ones influence disease development, although with a lower risk.

A different disease risk caused by the diverse DRB1*03-bearing haplotypes has also been described in type 1 diabetes (T1D). Baschal et al. reported the highest risk for the AH 18.2 and the lowest risk for AH 8.1 [15]. More powered studies are required to establish whether the AH 8.1 contributes to MS development. Our data discard a strong effect for AH 8.1, as the one observed for AH 18.2, and, consequently, our sample size could not be enough to obtain a significant result, although it might exist. The reported association between DRB1*03:01 and MS risk in some Northern-European populations [5], where the most common DRB1*03:01 haplotype is the AH 8.1, suggests that it could be contributing to disease risk.

The previously described association of MS and OCMB with *TNF* -376A seems to be due to the presence of this allele on the AH 18.2 and therefore, to the linkage disequilibrium with the causal variant/s present on that haplotype.

The association of AH18.2 and intrathecal anti-lipid IgM synthesis could have important clinical implications. The presence of those antibodies has been related to development of an aggressive MS [14], [16], [17] and it has been described that those patients could benefit of an early immunomodulatory treatment [18]. Moreover, a better response to treatment with interferon-beta has been described for patients lacking OCMB [19]. Therefore, MS patients carrying the AH18.2 deserve special attention. It must be underlined that only 4% of patients with that haplotype do not have intrathecal IgM. However, further research is needed since AH 18.2 appears in 12% of M+ individuals and additional factors must therefore contribute to IgM production.

MS is characterized by an extensive heterogeneity in terms of clinical features, pathogenesis or responsiveness to treatments. The mechanism underlying those differences is still uncovered, but a genetic contribution must exist. Therefore, the analysis of the overall MS patients can miss genetic variants relevant for certain groups, as it is proved in our work. On the contrary, the study of well-characterized subgroups seems to be a good strategy to further increase the number of genes involved in MS susceptibility, contributing to elucidate part of the “missing heritability” [20]. Despite the great advances in the genetics of MS, more than 50% of the disease heritability remains unsolved. Our results show an increase of the odds ratio as we define the best marker of the causal variant/s and the specific affected subgroup. Thus, from an initial OR = 1.52 conferred by DRB1*03:01, we moved to OR = 1.94 caused by the specific AH 18.2 to MS overall and to OR = 2.98 when we consider specifically M+ patients. Note that this effect is similar to the one caused by DRB1*15:01, the main risk factor to MS susceptibility. A parallel situation is expected for genetic variants affecting only subgroups of patients. According to our data, promising results can be expected from studying more homogeneous groups of MS patients.
